# Rapid Adaptation Often Occurs through Mutations to the Most Highly Conserved Positions of the RNA Polymerase Core Enzyme

**DOI:** 10.1093/gbe/evac105

**Published:** 2022-07-25

**Authors:** Yasmin Cohen, Ruth Hershberg

**Affiliations:** Rachel & Menachem Mendelovitch Evolutionary Processes of Mutation & Natural Selection Research Laboratory, Department of Genetics and Developmental Biology, the Ruth and Bruce Rappaport Faculty of Medicine, Technion-Israel Institute of Technology, Haifa 31096, Israel; Rachel & Menachem Mendelovitch Evolutionary Processes of Mutation & Natural Selection Research Laboratory, Department of Genetics and Developmental Biology, the Ruth and Bruce Rappaport Faculty of Medicine, Technion-Israel Institute of Technology, Haifa 31096, Israel

**Keywords:** rapid adaptation, bacterial evolution, evolutionary experiments

## Abstract

Mutations to the genes encoding the RNA polymerase core enzyme (RNAPC) and additional housekeeping regulatory genes were found to be involved in adaptation, in the context of numerous evolutionary experiments, in which bacteria were exposed to diverse selective pressures. This provides a conundrum, as the housekeeping genes that were so often mutated in response to these diverse selective pressures tend to be among the genes that are most conserved in their sequences across the bacterial phylogeny. In order to further examine this apparent discrepancy, we characterized the precise positions of the RNAPC involved in adaptation to a large variety of selective pressures. We found that RNAPC lab adaptations tended to occur at positions displaying traits associated with higher selective constraint. Specifically, compared to other RNAPC positions, positions involved in adaptation tended to be more conserved in their sequences within bacteria, were more often located within defined protein domains, and were located closer to the complex’s active site. Higher sequence conservation was also found for resource exhaustion adaptations occurring within additional housekeeping genes. Combined, our results demonstrate that the positions that change most readily in response to well-defined selective pressures exerted in lab environments are often also those that evolve most slowly in nature.

**Significance**

Bacteria are capable of adapting very rapidly to strong selective pressures. Here, we show that those sites that are involved in adaptation and therefore evolve most rapidly in response to specific selective pressures are surprisingly the same sites that evolve most slowly over longer evolutionary timescales. These results suggest that the mutations that are most adaptive in response to specific selective pressures are either limited from contributing to adaptation in most natural environments, or that if they do occur, are very specific to certain conditions and are therefore highly transient within natural bacterial populations.

## Introduction

Evolutionary experiments have been instrumental in enabling researchers to study evolution as it happens within controlled environments and particularly in enabling the study of bacterial adaptation (Kawecki, et al. 2012; Barrick and Lenski 2013; Katz, et al. 2021). Bacteria in particular are useful for evolutionary experiments, because they have short generation times, enabling to study their evolution over relatively large numbers of generations, in a relatively short amount of time. Many bacterial species can be frozen and later revived, allowing researchers to go “back in time” and compare an evolved strain to its ancestor. During evolutionary experiments, bacterial populations are exposed to specific selective pressures and the manner in which they adapt to these pressures is examined. The advent of next generation whole genome sequencing technologies enabled many studies that characterized the adaptive mutations that occur in response to specific selective pressures. Given that *Escherichia coli* is the most commonly used bacterial model organisms, a substantial fraction of such studies was carried out in *E. coli*.

Evolutionary experiments have highlighted the remarkable capability of bacteria to undergo relatively rapid adaptation. Such adaptation often occurs through mutations to very central housekeeping genes (reviewed in [Hershberg 2017]). The most obvious example of this trend is adaptations occurring within the RNA polymerase core enzyme (RNAPC) genes, *rpoB* and *rpoC*. The *rpoB* gene encodes the RNAPC’s β subunit, and *rpoC* encodes its β’ subunit. These two subunits occupy 80% of the total mass of the core enzyme and together form its active site (Sutherland and Murakami 2018). Mutations within *rpoB* and *rpoC* were shown to be involved in adaptation to a variety of selective pressures including exposure to lethal doses of antibiotics (Severinov et al. 1993; Reynolds 2000; Delgado et al. 2001; Srivastava et al. 2012; Degen et al. 2014), high temperatures (Tenaillon, et al. 2012), low nutrients (Conrad et al. 2010), exposure to radiation (Bruckbauer et al. 2019), and prolonged resource exhaustion (Avrani et al. 2017; Gross et al. 2020).

The fact that housekeeping genes such as the RNAPC tend to rapidly acquire adaptive mutations, altering their sequences, in response to a large variety of selective pressures, stands in apparent contrast to the high levels of conservation of these genes. Housekeeping genes in general and the RNAPC in particular tend to be extremely well conserved in their sequences, structure and function from bacteria to humans (Archambault and Friesen 1993; Zhang et al. 1999). This conservation is extensive enough to allow the bacterial RNA polymerase to serve as a model for understanding the basic principles at work in all cellular RNA polymerases (Borukhov and Nudler 2008). Within bacteria the sequences of *rpoB* and *rpoC* are conserved enough to enable their usage as a slowly evolving gene markers in the study of bacterial phylogeny (Lan et al. 2016).

Here, we characterize the positions of the RNAPC involved in known adaptations. We show that a unique set of RNAPC positions are involved in adaptation to different conditions, with very little overlap seen between different conditions. Furthermore, when positions involved in adaptation are combined across conditions, they tend to be even more conserved in bacteria, than other positions of the RNAPC and tend to be located more closely to the protein complex’s active site. When broken down by selective pressure these trends hold for positions involved in adaptation to antibiotic exposure, to prolonged resource exhaustion, to growth in minimal media, but not for positions involved in adaptation to high temperatures. Finally, we show that under resource exhaustion, adaptations occurring within non-RNAPC housekeeping genes also tend to fall within more conserved positions of these proteins.

## Results

### Little Overlap in the RNAPC Positions Involved in Adaptation to Various Selective Pressures

We carried out a literature survey to annotate protein positions involved in adaptation to a variety of selective pressures, within the RNAPC proteins RpoB and RpoC, in the model bacterium *E. coli*. This resulted in the identification of 140 positions (table 1).

**Table 1:** Summary of RNAPC Positions Involved in Rapid Adaptation

Selective Pressure	Strain	Number of Positions Involved in Adaptation	RpoB Positions Involved in Adaptation	RpoC Positions Involved in Adaptation	References
**Antibiotic exposure**	K-12 MG1655	51	146;148;509;511;512;**513**;**516**;**522**;526*;**529**;**531**;533;544;545;563;564;569;572*;574;675;**677**;687;1232;1255;**1275**;1278;1279;1285;1291;1296;1298;1315;1317;1320;**1322**;1325*;	345;690;697;**738**;748;758;763;775;**779**;780;**782**;783;**917**;931;**1354**;	(Severinov, et al. 1993; Reynolds 2000; Mukhopadhyay, et al. 2008; Wrande, et al. 2008; Srivastava, et al. 2012; Degen, et al. 2014; Field and Hershberg 2015)
**Prolonged resource exhaustion**	K-12 MG1655	16	814;1237;1244;1268;**1272**;1277;1321;1325*;	334;375;428; 434;469;504; 621;1357*;	(Avrani, et al. 2017; Gross, et al. 2020; Katz, et al. 2021)
**Growth at high temperatures**	K-12 MG1655	56	**84**;97;143;151;365;372; 375;539;553;556;566; 572*;664;**725**;**745**;**747**; 758;760;806;866;948;958;960;965;**966**;967; 1014;1078;1081;1210;1236;1243;1245;1250;1297;1316;**1323**;1330;	106;218;223; 290;369;373; 493;511;825; 833;866;903; 1099;1127; 1130;1315; 1336;1357*;	(Tenaillon, et al. 2012)
**Radiation**	K-12 MG1655	2	72;	1172;	(Bruckbauer, et al. 2019)
**Glucose minimal media**	K-12 MG1655	5	546;671;672;673;1100;		(Conrad, et al. 2010)
**Glycerol**	K-12 MG1655	2	562;	750;	(Herring, et al. 2006)
**Deletion of major metabolic gene**	K-12 MG1655	2	1242;	1174;	(Charusanti, et al. 2010)
**^13^C glucose**	K-12 MG1655	2	657,1189		(Sandberg, et al. 2016)
**Heavy metal**	K-12 MG1655	3	520;526*;	395;	(Graves, et al. 2015)
**Atmospheric pollution**	BW25113	1	12;		(Zhang, et al. 2019)
**Resource exhaustion starting with RpoS mutant**	K-12 MG1655	1		494;	(Nandy, et al. 2020)
**Acidic conditions**	K-12 3110	3	679	507,774	(Harden, et al. 2015)

Note.—Positions marked in bold undergo at least two different types of mutations in response to a single selective pressure. Positions marked by an asterisk are involved in adaptation to more than a single selective pressure.

It is important to distinguish between antibiotic resistance and other types of adaptation, as in the case of antibiotic resistance the reason for the occurrence of the mutations within the RNAPC is different. Specifically, antibiotic resistance adaptations occur within the RNAPC genes, because the antibiotics they confer resistance to themselves target those genes. Mutations that confer resistance are those that alter the structure of the protein so that the antibiotic can no longer effectively bind it (Spratt 1994). In contrast, an RNAPC mutation that provides an advantage under, for example, prolonged resource exhaustion likely owes its adaptive effect to the effects it has on the function of the RNAPC in regulating gene expression. Strikingly, we observe very little overlap in the RNAPC positions involved in adaptation to different selective pressures. Of the 140 positions in our dataset of positions involved in adaptation, only one is involved in adaptation to two different non-antibiotic related selective pressures, and only three are involved in both antibiotic resistance and an adaptation to a second selective pressure.

### Adaptation Tends to Occur within More Conserved Positions of the RNAPC

Next, we examined whether the 140 RpoB and RpoC protein positions in which adaptations were found, differed in their levels of sequence conservation, compared to the remaining RpoB and RpoC positions. To do so, the *E. coli* K12 MG1655 RpoB and RpoC sequences were compared, using BLAST, at the protein level against a database of the full proteomes of 44,048 fully sequenced bacterial genomes. Only bidirectional best hits were maintained. Each of the identified RpoB and RpoC sequences was then realigned at the protein level against their *E. coli* K12 MG1655 ortholog, using the Needleman-Wunsch pairwise alignment algorithm, as implemented by the EMBOSS (European Molecular Biology Open Software Suite) needle program. This enabled us to compute the optimal alignment (including gaps) of each two sequences along their entire length. In order to avoid biases, resulting from over sampling of closely related bacterial strains with identical RNAPC genes, if two RpoB or two RpoC orthologs were found to be identical in their sequences, only one of the two was maintained. Finally, we filtered out alignments that had less than 30% overall sequence identity across their entire length. From the resulting 8,163 RpoB alignments and 7,727 RpoC alignments, we calculated the percentage of strains in which each position of the *E. coli* K12 protein sequence was conserved. We then compared levels of conservation, between the 140 positions that were shown to be involved in lab adaptation, and the remaining protein positions. This enabled us to demonstrate that positions in which adaptations are found tend to be more conserved than all remaining positions (fig, 1; Supplementary table S1, Supplementary Material online; *P* < 0.001 for both RpoB and RpoC, according to a one-tailed non-paired Mann–Whitney test).

**Fig. 1.**
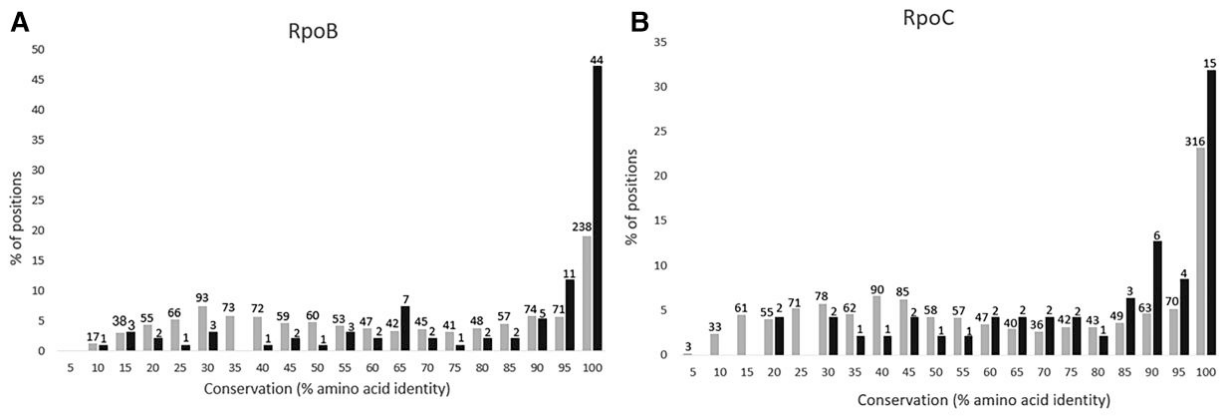
Positions in which known rapid adaptations occur tend to be more conserved than those in which no such adaptation is known. Depicted in each graph are the distributions of conservation levels of RpoB (*A*) and RpoC (*B*) positions, divided into positions in which known adaptive mutations were found to occur (black) and those in which no such adaptive mutations were yet identified (gray). Numbers above each bar indicate the numbers of positions falling within each conservation bin. Positions in which known adaptations occur are significantly more conserved for both RpoB and RpoC (*P* << 0.001).

For adaptation to antibiotic resistance, growth at high temperatures and prolonged resource exhaustion, sufficient positions are involved to test differences in conservation separately for each selective pressure. Adaptations to prolonged resource exhaustion and adaptations leading to antibiotic resistance occur at positions that were more conserved than positions at which no adaptations were found (*P* << 0.001 for adaptations occurring within RpoB and *P* < 0.008, for adaptations occurring within RpoC; Supplementary fig. S1, Supplementary Material online). In contrast no significant difference was found in the conservation of positions involved in adaptation to growth at high temperatures and positions with no observed adaptation (Supplementary fig. S1, Supplementary Material online). When all remaining positions, in which adaptations occur are combined and compared against positions with no known adaptations, a significant difference in conservation is observed within RpoB (*P* = 0.004). For RpoC, we cannot find such a significant difference, possibly due to a very small number of positions involving other adaptations found within RpoC (*n* = 7).

To make certain that our results were not somehow due to the specific way we measured conservation, we repeated these analyses using three additional metrices of conservation, implemented in (Capra and Singh 2007) (Materials and methods). The results reported above remain consistent using all three metrices (Supplementary table S2, Supplementary Material online).

The *Proteobacteria* phylum to which *E. coli* belongs is one of the most well studied of bacterial phyla. As a result, sequences belonging to this phylum are likely to be over-represented within our database. As this could potentially bias results, we aimed to verify that RNAPC positions tend to be more conserved across all phylogenetic distances. To do so, we separated the 8163 of RpoB and 7727 RpoC alignments we obtained, according to their percent identity, into 10% sized bins (eg. 90–100%, 80–90% etc…). We then examined whether positions in which adaptations were found in *E. coli* tended to be more conserved, than positions in which no adaptations were observed, based on each group of alignments separately. In the case of RpoC, positions involved in adaptation are significantly more conserved than remaining positions, for all phylogenetic distances (*P* < 0.05, for all comparisons; Supplementary fig. S2, Supplementary Material online). In the case of RpoB, this was true (*P* < 0.001; Supplementary fig. S2, Supplementary Material online) for all but the most closely related alignments (90–100% identity, *P* = 0.1237). Our results thus demonstrate that positions involved in adaptation tend to be more conserved than other positions, across all phylogenetic distances.

### Positions Involved in Adaptation Tend to Fall Within Defined Functional Domains

The Uniprot database (Ponting and Russell 2002; UniProt Consortium 2021) provides annotation of functional domains. We used these annotations to divide the positions of the RpoB and RpoC protein sequences into those that fall within a defined functional domain, and those that do not. In addition to tending to be more conserved, RNAPC positions at which adaptations occur also tend to more often fall within residues belonging to defined functional domains (Supplementary table S1, Supplementary Material online). This is true in general, when all sites are considered together (*P* << 0.001 for RpoB and *P* = 0.0011 for RpoC, according to a Mann–Whitney test), and is also true when we consider antibiotic resistance adaptations (*P* << 0.001 for RpoB and *P* = 0.0085 for RpoC) and resource exhaustion adaptations (*P* << 0.001 for RpoB and *P* = 0.0016 for RpoC) separately. However, high temperature adaptations do not show a similar tendency to be enriched within defined functional domains (*P* = 0.1405 for RpoB and *P* = 0.2645 for RpoC).

### Positions Involved in Adaptation Tend to Be Located Close to the RNAPC Active Site

**Fig. 2.**
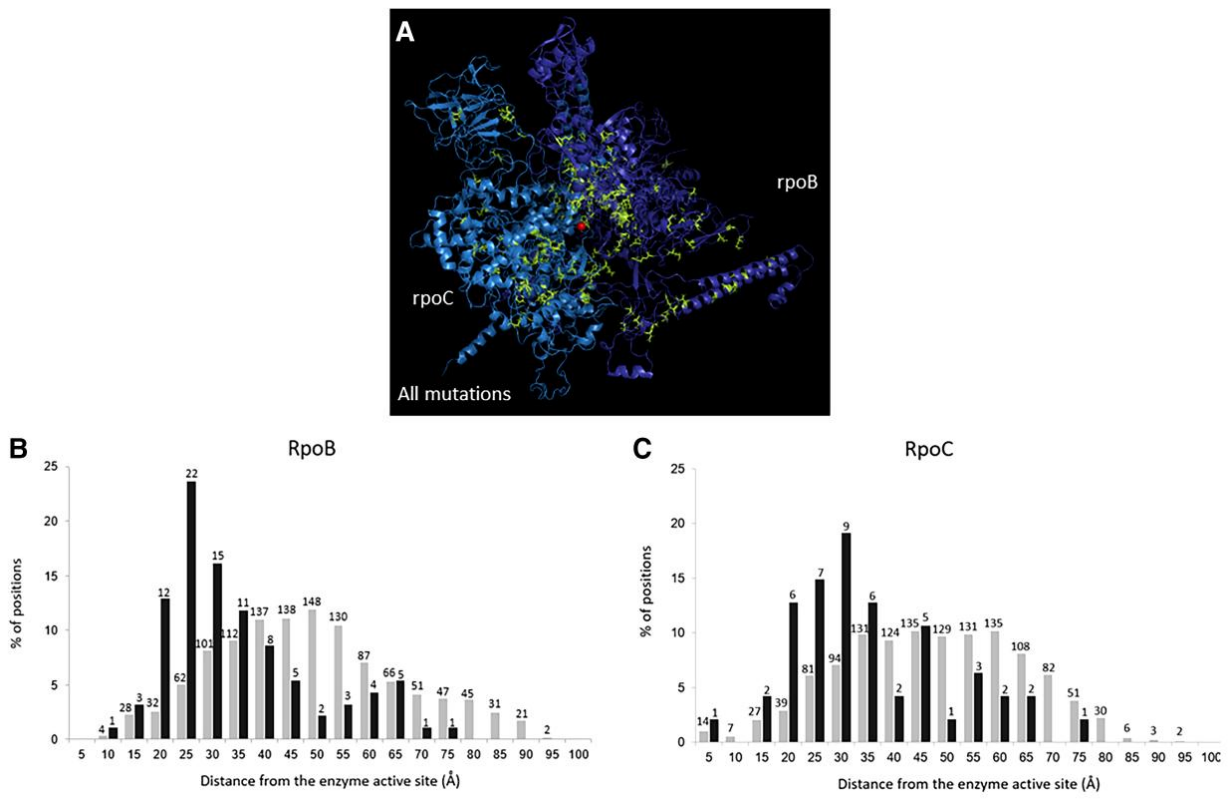
Positions involved in adaptation tend to be located closer to the RNAPC active site. (*A*) The solved protein structure of the RpoB-RpoC complex is presented (PDB accession 3LU0), with positions in which adaptations occur marked in green. Positions of both RpoB (*A*) and RpoC (*B*) at which known adaptations occur (black bars) tend to be located significantly (P << 0.001) closer to the protein complex’s active site, relative all other positions (gray bars). Numbers above each bar indicate the numbers of positions falling within each distance bin.

In order to further characterize the RNAPC positions involved in adaptation, we located them on the RpoB and RpoC complex solved protein structure (Protein Data Bank; 3LUO) (Opalka, et al. 2010) (fig. 2A). In general, positions involved in adaptation tended to be closer to the enzyme’s active site (as defined in its PDB structure) than other positions (*P*<<0.001, for both RpoB (fig. 2B) and RpoC (fig. 2C), and Supplementary table S1, Supplementary Material online). When considered separately, resource exhaustion, minimal media and antibiotic resistance adaptations tend to be located closer to the complex’s active site than positions that are not involved in adaptation (*P* < 0.001, for all comparisons). At the same time, in agreement with their lower levels of conservation and lack of tendency to be enriched within functional domains, positions involved in adaptation to high temperatures do not display a strongly significant enrichment for proximity to the active site (*P* = 0.0253 for RpoB and *P* = 0.3509 for RpoC).

### Excluding High Temperature Adaptations, Positions Involved in Adaptation to the Same Condition Tend to Be Clustered on the Protein Structure

**Fig. 3:**
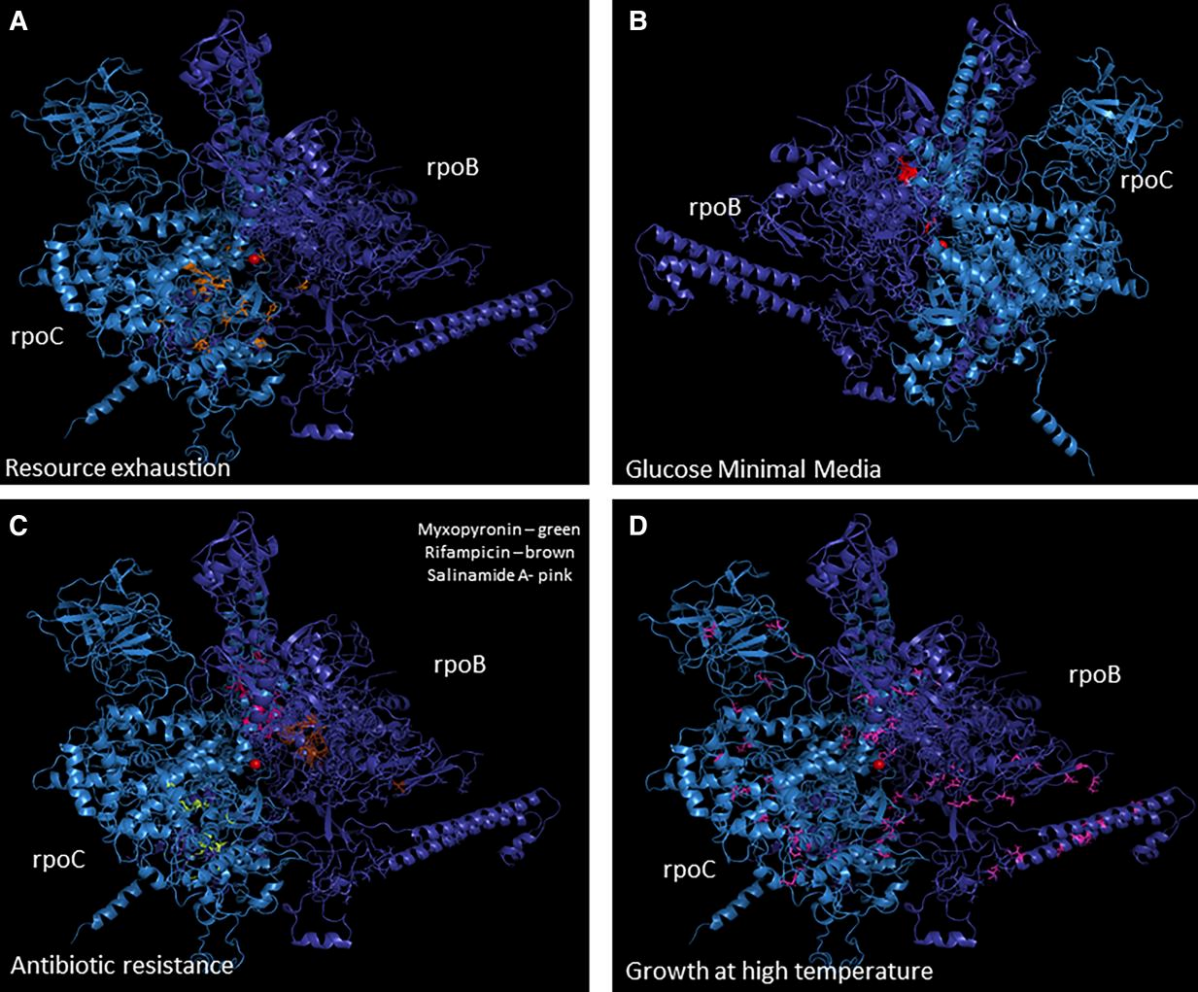
Locations of known adaptations on the solved protein structure of the RpoB-RpoC complex. The RpoB-RpoC protein structure was taken from the PDB accession 3LU0. Positions where known rapid adaptations occur marked on the structure: (*A*) Prolonged resource exhaustion adaptations. (*B*) Glucose minimal media adaptations. (*C*) Antibiotic resistance adaptations. (*D*) Growth at high temperature adaptations.

Resource exhaustion adaptations (Supplementary fig. 3A, Supplementary Material online) and minimal media adaptations (fig. 3B) tended to each separately cluster onto distinct close regions of the protein structure. Similarly, as expected, positions involved in adaptation to acquire resistance to the same antibiotic, also tend to cluster together (fig. 3C). In contrast, positions involved in adaptation to growth at high temperatures are more dispersed over the entire RpoB and RpoC complex structure (fig. 3D).

### Resource Exhaustion Adaptations within Additional Genes, Also Tend to Occur Within More Conserved Positions

We have been studying *E. coli* adaptation under prolonged resource exhaustion. In our experiments we found very high levels of convergence, with mutations often occurring within the same loci, across independently evolving populations (Avrani, et al. 2017; Katz, et al. 2021). Such convergence is widely considered to be a signal of adaptation. 19 genes (other than *rpoB* and *rpoC*) were identified in which mismatch mutations occurred across all five of our populations, indicating that these mutations are adaptive under resource exhaustion. These genes included *rpoA*, which encodes the alpha subunit of the RNAPC, *rpoD* that encodes the Sigma70 subunit of RNA polymerase and many additional global regulators of gene expression (Supplementary table S3, Supplementary Material online). To examine whether the positions of these genes at which convergent mutations occurred tend to be more conserved than other positions of these genes, we first carried out BLAST searchers, using each gene as a query and requiring bi-directional best hits, as done before for RpoB and RpoC. Bacterial strains for which we could not find an RpoB or RpoC ortholog were removed from consideration, as the RNAPC is known to be present across bacteria and strains that do not carry these genes are suspect as being poorly sequenced. Alignments were then refined using the Needleman-Wunsch algorithm, with at least 30% identity required, and identical alignments were clustered together into a single alignment. The numbers of bacterial strains in which we found each gene initially, and the number of ultimate alignments we were left to work with in the end are summarized in Supplementary table S3, Supplementary Material online.

**Fig. 4:**
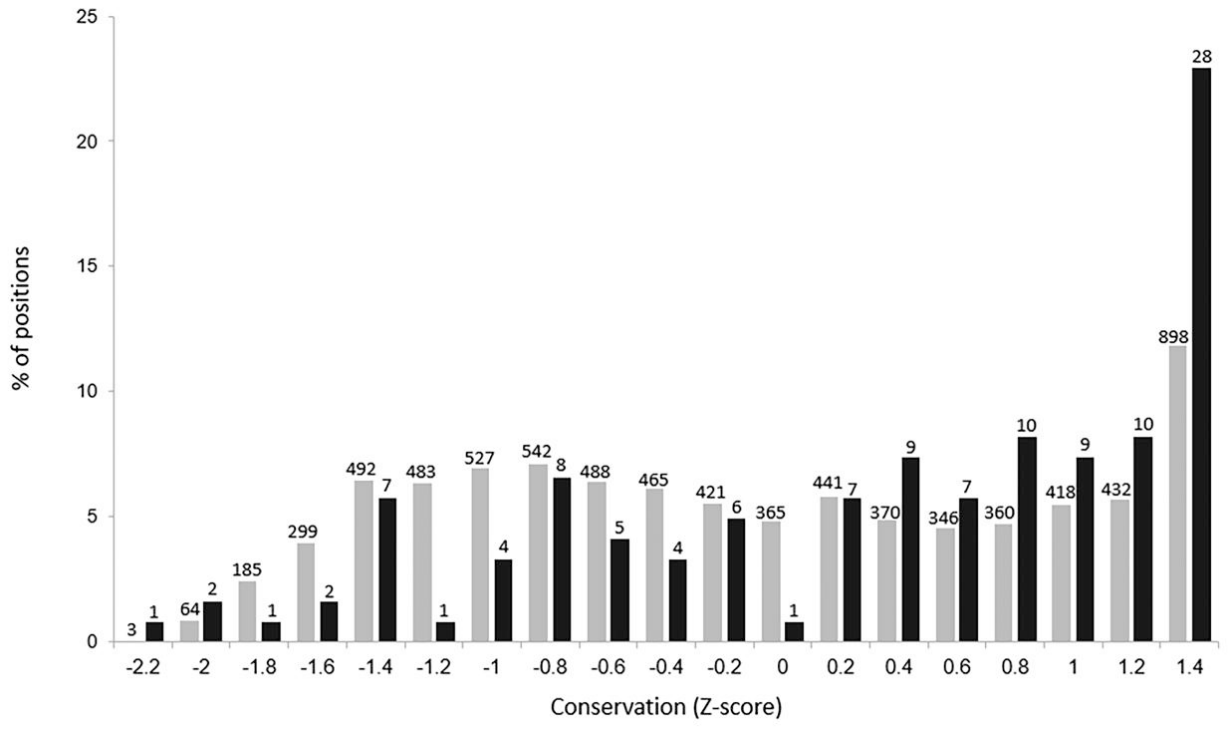
Adaptive mismatch mutations occurring within additional genes also tend to occur within more conserved positions of the proteins in which they occur. For this analysis, 19 genes found to be involved in adaptation to prolonged resources exhaustion were analyzed. Conservation levels of each position within these genes were normalized in order to enable the combination of data from all 19 genes. Depicted are the distribution of conservation levels of positions, divided into those in which resources exhaustion adaptations were identified (black), and all remaining positions (gray).

In contrast to RpoB and RpoC, here, for each gene, only a handful of positions were predicted to be involved in adaptation. In order to obtain sufficient power to examine whether positions likely involved in adaptation tended to be more conserved, we had to therefore combine data across the genes. To do so, while not biasing our results due to differences in overall conservation between the proteins, we normalized within each gene the calculated levels of conservation of each position by calculating a Z-score (Supplementary table S4, Supplementary Material online; see Materials and Methods). Once conservation levels were normalized, they could then be combined across genes. We found that the positions within convergently mutated genes, in which resource exhaustion mutations occurred, were significantly more conserved than remaining positions within the same genes (*P* << 0.001, according to a one-tailed nonpaired Mann–Whitney test; figure 4).

## Discussion

Our results demonstrate that adaptation often occurs within sites that are most conserved over larger evolutionary timescales. Moreover, the structural location of the sites involved in adaptation further suggests that these are likely constrained sites, as they tend to be located within defined functional domains and close to the RNAPC’s active site. Adaptations to high temperatures behave differently than this trend, as the positions in which they occur do not display higher conservation, are not clustered on the RNAPC structure, and are not enriched within functional domains or in close proximity to the complex’s active sites. For individual conditions to which only a few RNAPC adaptations were characterized, we cannot know whether adaptations behave more like the general trend we observed, or more like what is seen for high temperature adaptations. We are thus not claiming that our results hold for all, or even most lab conditions. However, they do hold for most currently characterized RNAPC adaptations, and for three out of four conditions for which sufficient adaptive sites are currently available for separate analyses.

The occurrence of many adaptations within the most highly conserved positions of generally highly conserved genes raises a conundrum: How is it possible for these positions to change so rapidly in response to a variety of selective pressures, yet remain so highly conserved over longer evolutionary time scales? The answer to this question may relate to pleiotropy (Cooper and Lenski 2000; MacLean, et al. 2004; Kvitek and Sherlock 2013). The specific changes to the RNAPC and additional master regulatory genes that are adaptive under a specific condition may have strongly deleterious pleiotropic effects under many, if not all other conditions. After all, master regulators in general, and the RNAPC in particular regulate the expression of large chunks of the transcriptome. In lab experiments, bacteria are generally exposed to relatively simple, strong and constant selective pressures. The selective pressures faced within more natural environments are likely far more complex, with several different factors exerting contradictory pressures simultaneously and/or with selective pressures that change with time. Adaptations of the kind that arise so easily during lab evolution may not be so easily permitted within natural environments, due to their pleiotropic effects. Additionally, if such adaptations do occur in response to a specific set of conditions, they may prove to be highly transient, rapidly decreasing in frequency once conditions change. Supporting this, we recently demonstrated such transience of RNAPC adaptations arising under resource exhaustion. We showed that bacteria exposed to prolonged resource exhaustion adapt via mutations to the RNAPC, but that these mutations do not tend to fix across an entire population (Avrani et al. 2020). Since these mutations carry pronounced costs to fitness under conditions of resource abundance and rapid growth, once bacteria are transferred into fresh media, rare clones that do not carry an RNAPC adaptation outcompete the clones that do carry these adaptations, leading to rapid reductions in the frequencies the RNAPC adaptations (Avrani et al. 2020). If RNAPC adaptations are in general highly transient, it may explain why the sites in which they occur can remain largely conserved, when one examines longer evolutionary timescales.

We find remarkably little overlap in the positions of the RNAPC involved in adaptation to various selective pressures, indicating that different selective pressures demand different specific changes to the RNAPC. While future discovery of additional sites involved in adaptation, may lead to the discovery of higher overlap, the near lack of overlap we observe is likely to be indicative of a general trend, given the large number of sites already included in our analyses. We further find that for most conditions, sites involved in adaptation tend to be clustered on the protein structure. Combined, these results strongly suggest that for many conditions, very specific changes to the RNAPC are adaptive.

The specificity of sites involved in adaptation under many selective pressures may suggest that adaptation to each pressure occurs through changing a specific function of the RNAPC, different from the function changed in response to a different selective pressure. In other words it is possible that the reason that specific positions are involved in adaptation to condition A, while different ones are involved in adaptation to condition B, is that the condition A adaptations drive specific gene expression changes adaptive under condition A, while condition B adaptations drive different changes to gene expression, adaptive under condition B. A second possible reason for the specificity of the adaptive mutations is that while the same ultimate adaptive outcome is always reached, the way to reach that outcome is condition dependent. In other words, the adaptive outcome may involve the same specific change to transcriptional kinetics, under both conditions A and B. However, due to changes in the structure of the transcriptional regulatory network under the different conditions, inducing the adaptive end result might involve different mutations in condition A, compared to condition B. Finally, high levels of specificity may also be explained not through the need to change a specific function, but through the need to prevent antagonistically pleiotropic effects (Cooper and Lenski 2000; MacLean, et al. 2004; Kvitek and Sherlock 2013). For example, a mutation may have an adaptive effect under both conditions A and B, but also have negative effects, unique to condition B. Such an adaptive mutation will only be allowed to occur under condition A and not under condition B, leading to specificity. These three explanations are by no means mutually exclusive. It is possible that they work in combination to explain the observed specificity of adaptive positions. For example, it is possible that certain positions are involved in adaptation under one condition, but not under another due to antagonistic pleiotropy, while other positions tend to be specific to a single condition, because they only drive beneficial changes to gene expression under that condition.

Some clues regarding the consequences of the RNAPC adaptive mutations may be gleaned from their location on its structure. In the case of antibiotic resistance adaptations, the reasons for their location are easy to predict: Resistance adaptations will likely occur within the same region of the complex that the antibiotic binds, and work by reducing the ability of the antibiotic to bind its target. When it comes to other types of adaptations it is less straight forward to predict their precise adaptive effect. Conrad *et al*. revealed the adaptive role of minimal media RNAPC adaptations in altering transcriptional kinetics, by decreasing the longevity of open complex (Conrad et al. 2010). Here, we found that resource exhaustion RNAPC adaptations are located within the complex’s clamp domain, which has been implicated in involvement in several crucial aspects of the transcription process, including transcription initiation and elongation (Duchi et al. 2018).

Mutations to the RNAPC involved in adaptation to growth at high temperatures, behave differently than those involved in adaptation to other conditions. While adaptation to high temperatures appears to occur within the RNAPC in a highly convergent manner, many different specific mutations were found to occur across independently evolving populations (Tenaillon et al. 2012). This stands in contrast adaptation to prolonged resource exhaustion, where the same specific sites tend to be mutated across independently evolving populations (Avrani et al. 2017; Katz et al. 2021). The many high temperature RNAPC adaptations do not tend to occur within significantly more conserved positions, are not enriched within known functional domains or in proximity to the active site, and they are not clustered together on the complex’s structure. This appears to suggest that unlike adaptations to other conditions, high temperature adaptations may be acting through a less specific mechanism, affecting some more general trait of the RNAPC, that does not require changes to very specific sites, located at the heart of the complex. Intriguingly, studies into the effects of RNAPC high temperature adaptations suggested that these adaptations change the expression of hundreds of genes back towards the transcriptional program of pre-stressed bacteria (Rodriguez-Verdugo et al. 2016). It will be interesting to understand how this can be achieved by such a large variety of non-specific mutations, located all over the RNAPC’s structure.

Another possibility is that the reason for the differences observed between high-temperature adaptations and adaptations to resource exhaustion, growth in minimal media, and antibiotic exposure has less to do with the different responses to these selective pressures and more to do with experimental setup. Various evolutionary experiments tend to be carried out differently. This means that in addition to the differences between experiments in the type of selective pressure that is implicitly studied, there may also be additional differences that are less obvious and that could influence evolutionary outcomes. For example, we have previously shown that different RNAPC positions are involved in adaptation to resource exhaustion, as a function of the growth volume used for the experiments (Gross et al. 2020). The high-temperature evolutionary experiments (Tenaillon et al. 2012) were initiated from a clone that was pre-adapted for 2000 generations for growth within the experimental media, at 37°C, while other experiments did not so pre-adapt their ancestral clones. While we do not see this as very likely, it is possible that this pre-adaptation somehow changed the nature of the adaptive mutations seen in the experiment, leading to some of the differences seen between high-temperature adaptations and other adaptations.

In conclusion, our results reveal that under lab-evolution, many adaptations occur within the most conserved and constrained positions of genes that are in themselves highly conserved and constrained. We show such higher conservation and constraint of sites involved in adaptation to three of four conditions, for which sufficient sites were identified to be considered separately (resource exhaustion, growth in minimal media and antibiotic exposure). At the same time sites involved in adaptation to high temperatures show no enhanced pattern of conservation and constraint. These observations leave many questions open: 1) Under what conditions will adaptation tend to occur at more or less conserved sites? 2) Why is there so little overlap observed between the sites involved in adaptation to various conditions? Is this indeed due to specific sites being involved in adaptation to one condition but not another? If so, what explains such specificity? 3) Why are adaptations that arise so readily in the lab not generally seen in nature? Is it due to specificity of adaptive effects to very narrow conditions? Do such adaptive mutations never occur in nature, or do they occur under specific conditions, but rapidly reduce in frequency once conditions change? Answering these questions will require further analyses. First, it will be very useful to identify additional adaptations within the RNAPC and other housekeeping genes. Second, it will be useful elucidate the mechanisms of action of such adaptive mutations and to quantify their fitness effects under various conditions. Finally, more sophisticated methods of metagenomics will be required to examine whether RNAPC variation does occur within natural environments as well, under specific conditions, and perhaps persists at lower frequencies that cannot be easily observed using regular approaches.

## Materials and Methods

### Datasets

*E. coli* K12 MG1655 protein sequences were downloaded from the National Center for Biotechnology Information database (May 2019) (NCBI Resource Coordinators 2018). The protein sequences of 44,048 fully sequenced bacterial strains were downloaded from the Ensembl database (March 2020) (Yates, et al. 2020).

### Identification and Pairwise Realignment of Orthologous Genes

To identify the orthologs of RpoB, RpoC and the additional examined genes, we carried out BLAST (Altschul et al. 1990) searches using each of the *E. coli* K12 MG1655 protein sequences as queries against the Ensmbl collection of proteomes. Best bi-directional hits were required in order to maintain an identified ortholog for further analyses. Each identified ortholog was re-aligned with the *E. coli* K12 MG1655 sequence, using the Needleman-Wunsch pairwise alignment algorithm, as implemented by the EMBOSS needle program (Rice et al. 2000) function in Biopython (Cock et al. 2009). This enabled us to compute the optimal alignment (including gaps) of each two sequences along their entire length. Alignments that had less than 30% overall sequence identity across their entire length were removed from consideration. To avoid biases stemming from over-representation of certain closely related groups of strains within our dataset, with identical protein sequences, identical sequences we combined into a single representative. Supplementary table S3, Supplementary Material online summarizes the number of alignments obtained following this procedure, for each of the studied genes.

### Protein Sequence Conservation Estimation

Based on the obtained pairwise sequence alignments, we calculated for each position the percentage of alignments in which the amino acid of the query sequence is identical to that of the *E. coli* K12 strain used in this study. In addition to this simple method of conservation assessment, we also utilized three metrices implemented by (Capra and Singh 2007) in the https://compbio.cs.princeton.edu/conservation/score.html webtool: 1) the Shannon entropy of residues (SE) metric; 2) the Shannon entropy of residue properties (also referred to as property entropy metric); and 3) the Jensen-Shannon divergence score. While two of these methods are arguably more sophisticated, all three methods have the disadvantage of relying on multiple sequence alignments (MSA), which are per-definition less reliable than pairwise alignments calculated using dynamic programing. To generate an MSA of RpoB and RpoC orthologs, we drew at random 1000 RpoB orthologs, and 1,000 RpoC orthologs; out of all the orthologs, we identified for each of the two genes. From these sequences (as well as the *E. coli* reference sequences), we generated an MSA using the online Clustal Omega tool (https://www.ebi.ac.uk/Tools/msa/clustalo/) (Madeira, et al. 2022)

### Z-Score Calculations

In order to be able to combine different genes that vary in the distributions of the conservation levels of their positions, we calculated for each gene separately a Z-score for each of its positions as:

$$Z=\frac{x-\mu }{\sigma }$$

Where: x denotes the percentage of strains in which that position is conserved, µ denotes the mean percentage conservation across all positions of that protein, and σ denotes the standard deviation around that mean. Z score values were then combined across genes allowing us to compare them between positions in which adaptations occurred and all other positions.

### Mapping RpoB an RpoC Positions onto the RNA Polymerase Molecular Structure

We mapped the RpoB and RpoC positions involved in adaptation onto the three-dimensional structure of the RNA polymerase complex of *E. coli* (Protein Data Bank; 3LUO) (Opalka, et al. 2010). The mutations were mapped and visualized using PyMol (The PyMOL Molecular Graphics System, Version 2.4, Schrödinger, LLC) and Pyrosetta (Version 2.6) (Chaudhury, et al. 2010). The distance between the residues and the active site was measured with the PyMol distancetoatom function.

In order to classify positions according to whether they fall within an annotated protein domain, the domain annotation was taken from UniPort Knowledgebase (UniProt Consortium 2021), entries P0A8V2 (RpoB) and P0A8T7 (RpoC).

## Supplementary Material

evac105_Supplementary_Data

## Data Availability

All data used in this study are publicly available, with sources and accession numbers cited where relevant.
