# Supplementary material for: Rapid Adaptation Often Occurs through Mutations to the Most Highly Conserved Positions of the RNA Polymerase Core Enzyme
Source: Genome Biol Evol. 2022 Jul 25;14(9):evac105. doi: 10.1093/gbe/evac105 (PMC9459352; doi:10.1093/gbe/evac105)
Supplement: evac105_Supplementary_Data [file evac105_supplementary_data.zip › tableS2.pdf]

Table S2. Significance (P-values) for higher conservation of positions involved in adaptation, according to three MSA-based conservation estimates

RpoB

	**Shannon entropy of residues**	**Shannon entropy of residue properties**	**Jensen-Shannon divergence score**
All positions involved in adaptation combined	1.51E-15	7.82E-13	6.62E-16
Positions involved in adaptation to high temperatures	1.54E-02	3.31E-02	1.42E-02
Positions involved in adaptation to antibiotic exposure	2.17E-12	5.03E-11	1.35E-12
Positions involved in adaptation to prolonged resource exhaustion	3.90E-05	2.29E-04	2.10E-05
Positions involved in adaptation to all remaining conditions	1.30E-05	1.67E-04	1.10E-05

RpoC

	**Shannon entropy of residues**	**Shannon entropy of residue properties**	**Jensen-Shannon divergence score**
All positions involved in adaptation combined	1.83E-04	3.72E-04	2.77E-04
Positions involved in adaptation to high temperatures	1.22E-01	9.54E-02	1.83E-01
Positions involved in adaptation to antibiotic exposure	1.10E-03	3.09E-03	8.10E-04
Positions involved in adaptation to prolonged resource exhaustion	1.34E-04	1.21E-04	1.70E-04
Positions involved in adaptation to all remaining conditions	6.80E-01	7.95E-01	6.59E-01
