# Supplementary material for: Rapid Adaptation Often Occurs through Mutations to the Most Highly Conserved Positions of the RNA Polymerase Core Enzyme
Source: Genome Biol Evol. 2022 Jul 25;14(9):evac105. doi: 10.1093/gbe/evac105 (PMC9459352; doi:10.1093/gbe/evac105)
Supplement: evac105_Supplementary_Data [file evac105_supplementary_data.zip › tableS3.pdf]

Table S3: Number of alignments left after filtration

Gene	Number of alignments Prior to filtration	Number of alignments following filtration
asmA	16,748	966
crp	31,745	781
cytR	28,755	820
dcuA	43,985	1652
deoR	14,485	730
dppA	17,556	2,175
fadR	11,916	648
glpF	34,254	2380
gltS	21,596	1,707
kgtP	20,173	2,407
oppA	21,726	1,993
paaX	17,384	720
prc	34,870	2516
putP	23,971	3,148
rimJ	28,682	1565
rpoA	43,375	4,457
rpoD	42,687	5,468
sstT	7,992	977
sucC	32,306	4,722
