# Supplementary figures and images for: Rapid Adaptation Often Occurs through Mutations to the Most Highly Conserved Positions of the RNA Polymerase Core Enzyme
Source: Genome Biol Evol. 2022 Jul 25;14(9):evac105. doi: 10.1093/gbe/evac105 (PMC9459352; doi:10.1093/gbe/evac105)

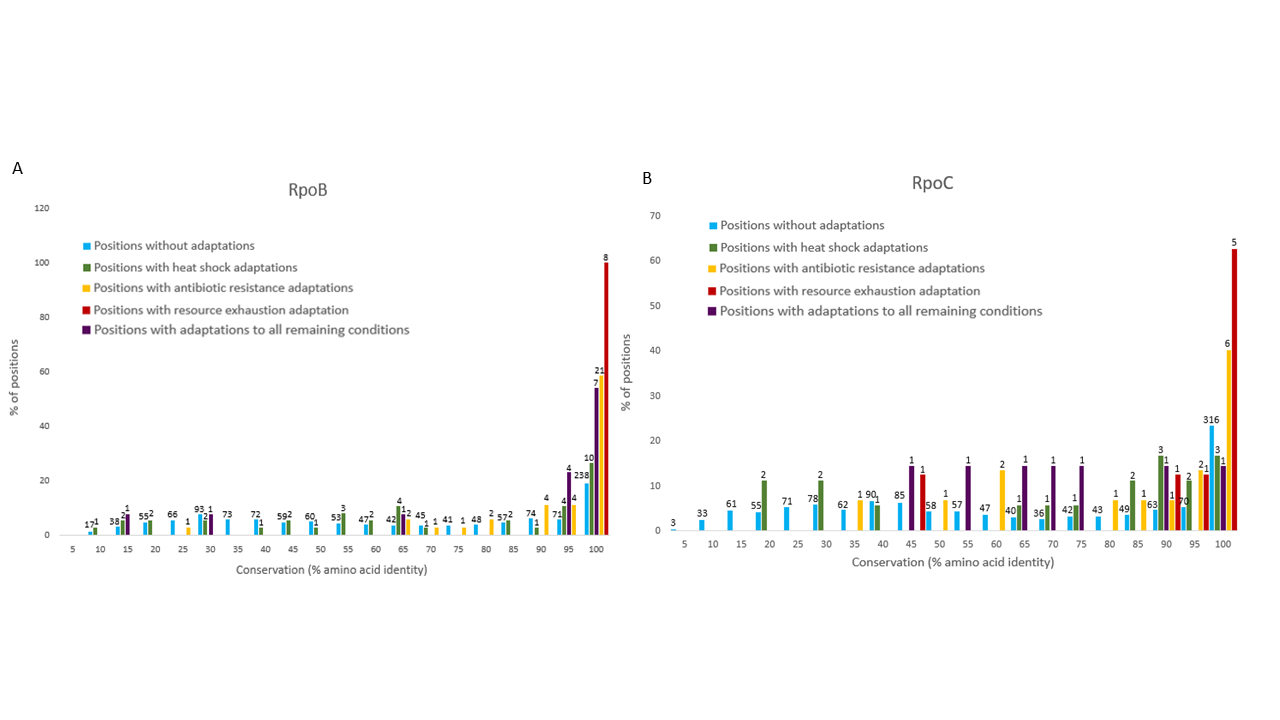

Supplement: evac105_Supplementary_Data [file evac105_supplementary_data.zip › figureS1.tif]

# rpoB

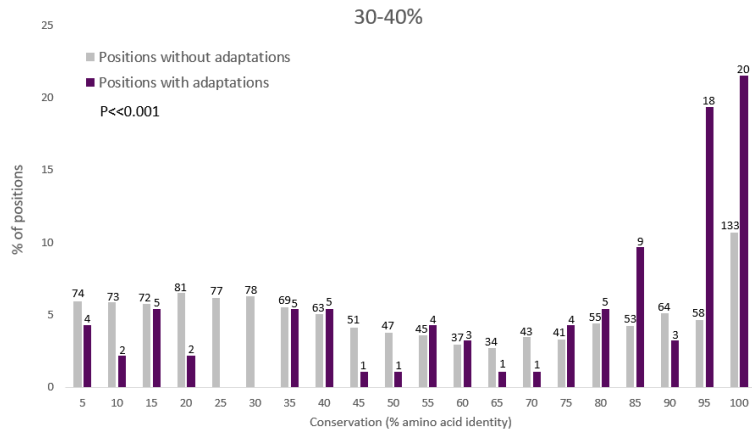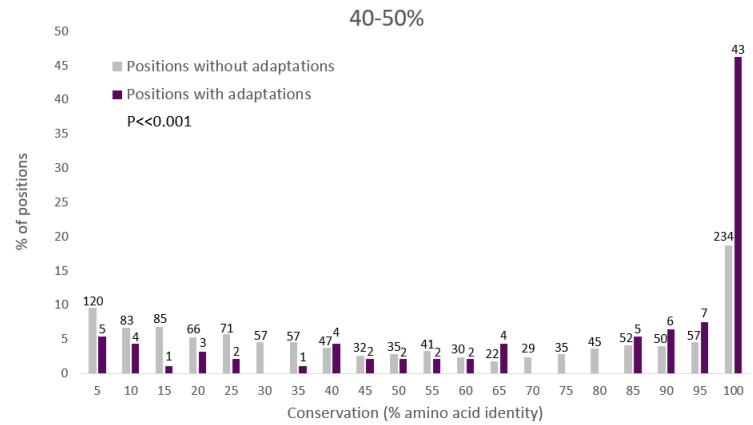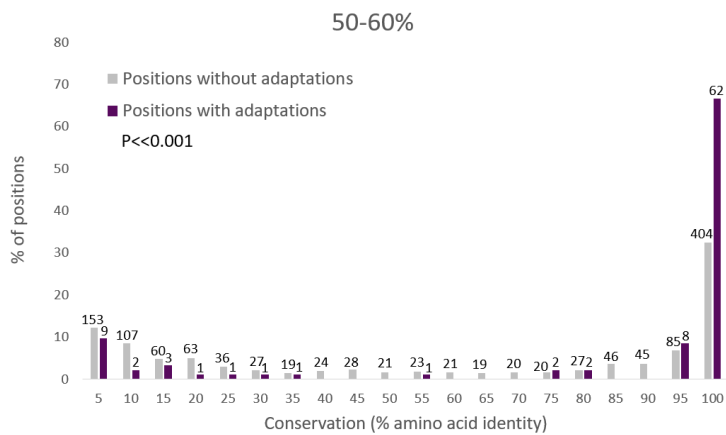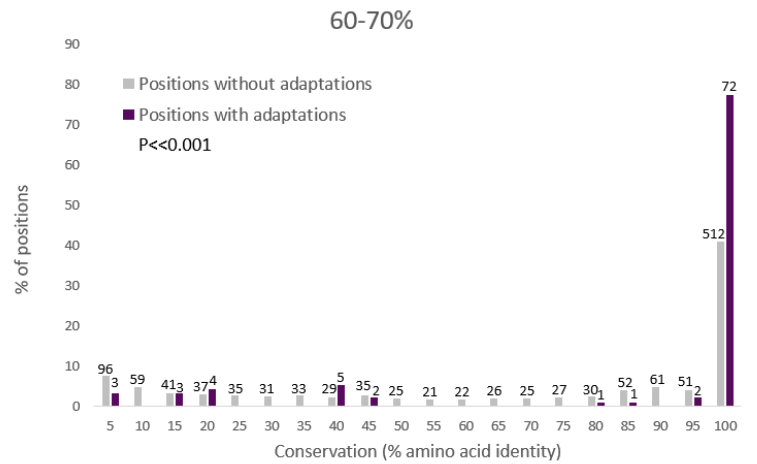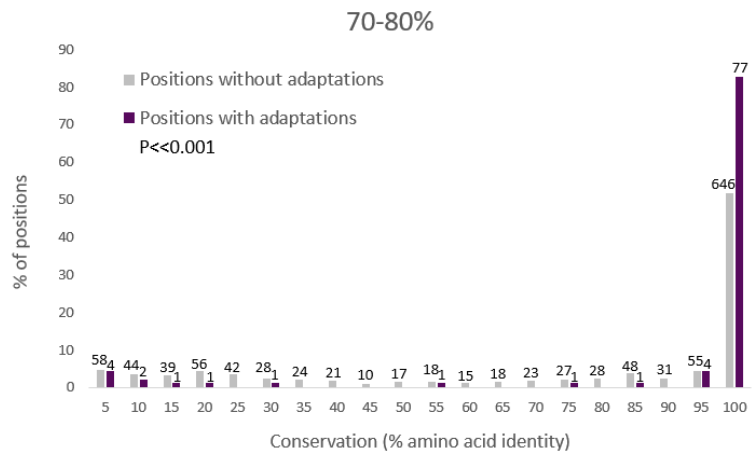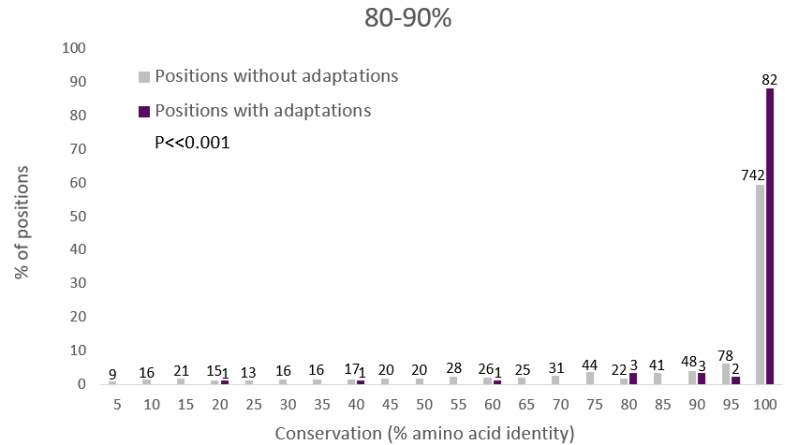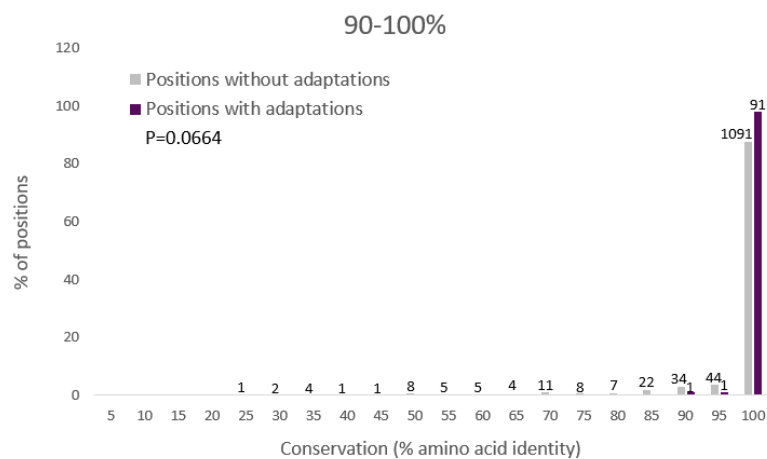

# rpoC

30-40%

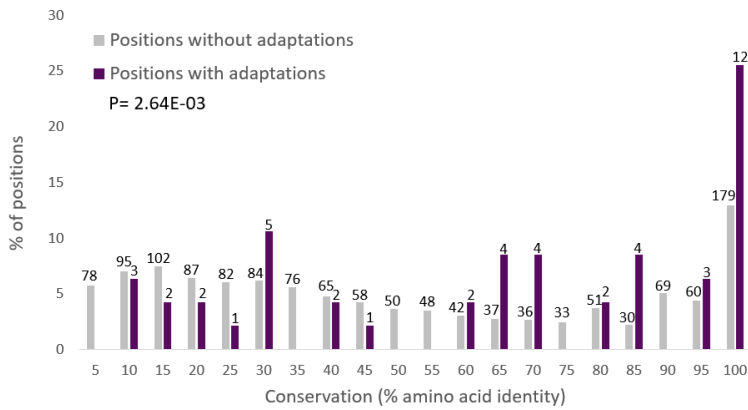

40-50%

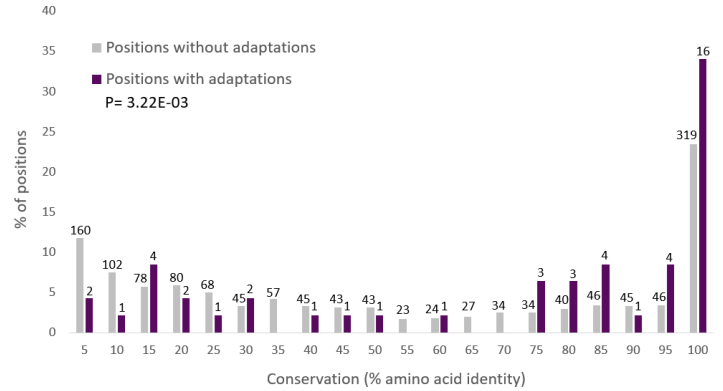

50-60%

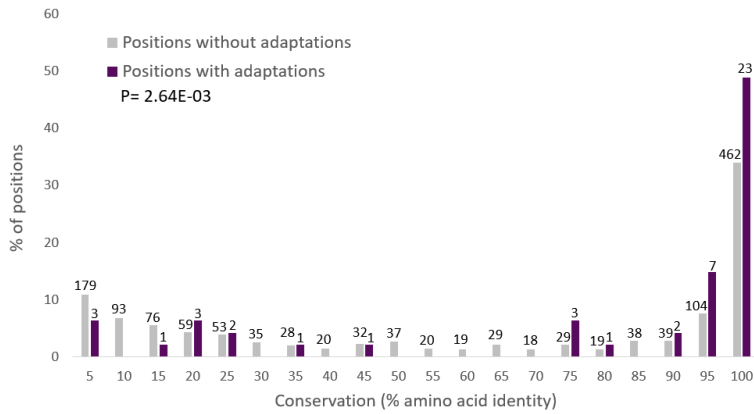

60-70%

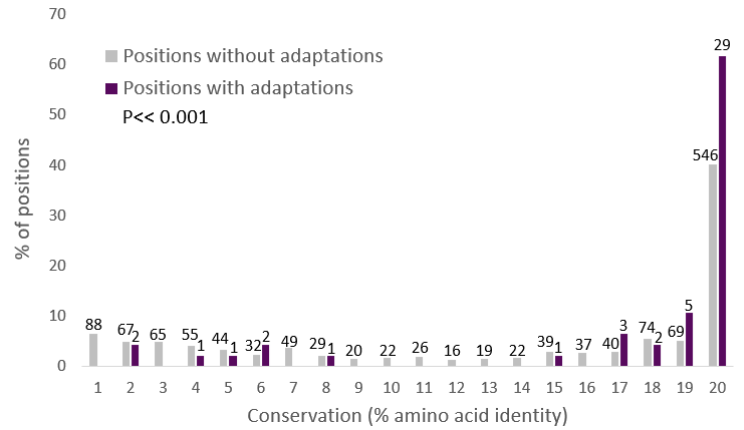

70-80%

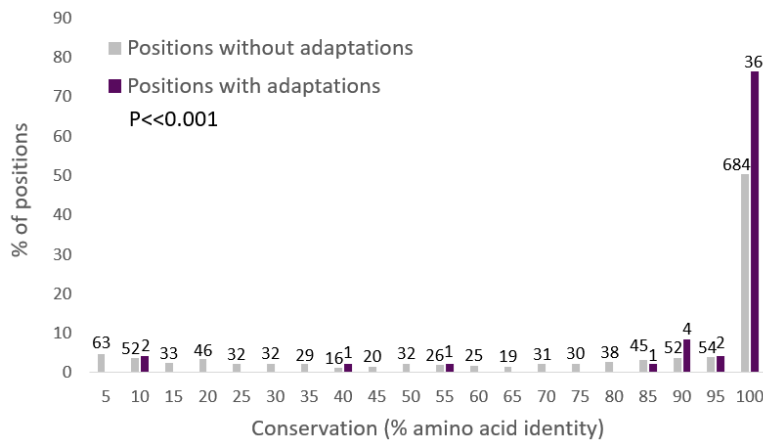

80-90%

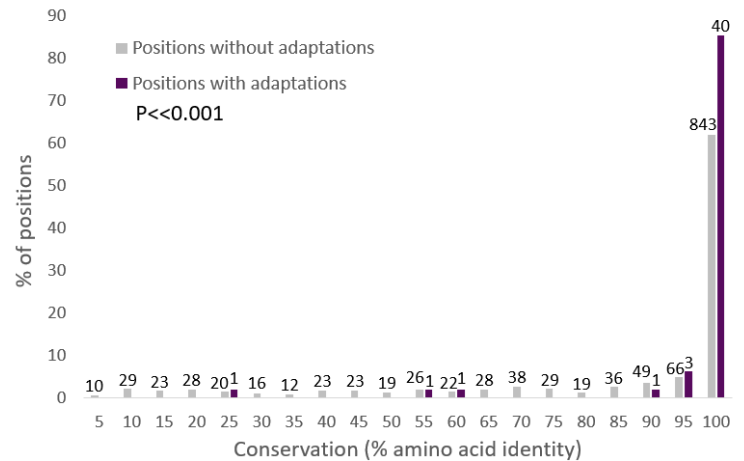

90-100%

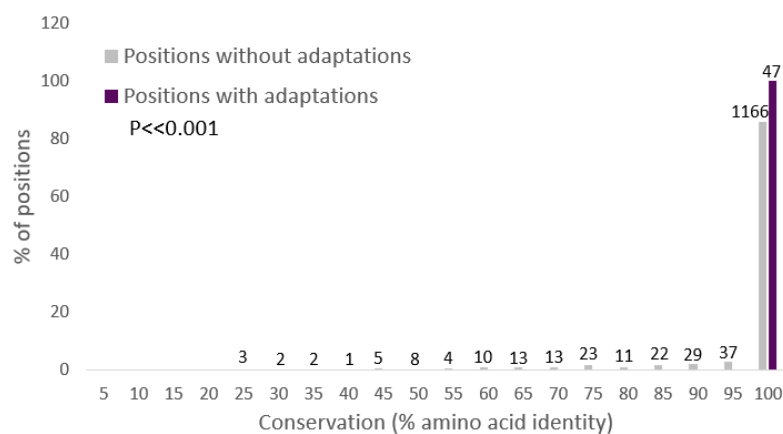

Supplement: evac105_Supplementary_Data [file evac105_supplementary_data.zip › FigureS2.pdf]
